# Dual-Task Interference Increases Variability in Sub-Second Repetitive Motor Timing

**DOI:** 10.3390/jfmk10040366

**Published:** 2025-09-25

**Authors:** Ivan Šerbetar, Asgeir Mamen

**Affiliations:** 1 Department of Kinesiology, Faculty of Teacher Education, University of Zagreb, 10000 Zagreb, Croatia; 2 School of Health Sciences, Kristiania University College, 0153 Oslo, Norway; asgeir.mamen@kristiania.no

**Keywords:** motor timing, dual-task performance, cognitive load, temporal variability, applied motor control, young adults

## Abstract

**Objectives:** Sub-second motor timing is critical for skilled performance in domains such as sport, music, and safety-critical multitasking; however, its robustness under cognitive load remains unresolved. Dual-task paradigms offer a method to test whether attentional demands selectively disrupt temporal precision. This study intended to investigate the effects of cognitive load on rhythmic finger tapping at a sub-second interval. **Methods:** A sample of 103 college students (19–25 years) performed a synchronization–continuation tapping task at 500 ms intervals under single- and dual-task conditions across five trials. The dual-task condition included a distracting letter-span task imposing working memory load. Inter-response intervals (IRIs), their variability (IRI SD), and accuracy (AI) were analyzed using linear mixed-effects models. **Results:** Tapping intervals were consistently shorter than the 500 ms target by approximately 70 ms in both conditions, showing anticipatory mechanisms that remained stable under cognitive load. Mean accuracy did not vary between single- and dual-task conditions. By contrast, temporal variability was significantly higher in the dual-task condition, reflecting diminished trial-to-trial consistency. These effects continued throughout trials and were supported by model estimates, which indicated robust between-subject variability but selective disruption of consistency rather than mean performance. **Conclusions:** Dual-tasking selectively hinders temporal stability in sub-second motor timing while ensuring that the reproduction and accuracy of the mean interval remain unchanged. This pattern supports dual-process accounts of timing, suggesting distinct roles for predictive control and attentional allocation. The results have applied relevance for situations requiring precise rhythmic performance under cognitive load, including sports, ensemble music, and safety-critical tasks.

## 1. Introduction

Human behavior always occurs within a specific time and space. Therefore, the ability to manage time cognitively is a fundamental human skill. While time is not a sensory modality like vision or audition but an essential dimension of sensory data—much like space—it is helpful to differentiate between sensory timing, which includes analyzing temporal relationships in the external world, and motor timing, which involves enforcing temporal structure through action, recognizing that many tasks contain elements of both [1]. The word *timing* can have the connotation of either the duration of an event or when an event happens [2]. Motor timing refers to the timing-related features of behavioral output, including the temporal coordination of movements, speech, or music.

These activities share a dependence on the sub-second timescale (hundreds of milliseconds), which is fundamental not only to locomotion but also to industrial operations (e.g., assembly line work) and athletic-specific movements. In sports such as basketball or soccer, rhythmic actions like dribbling or coordinated running steps must be maintained while simultaneously dividing attention to tactical decisions [3].

An intrinsic rhythmic structure defines a wide range of sports tasks as well. In addition to locomotor activities such as walking, running, cycling, or rowing, many other movements—such as jumping rope, swimming strokes, or dribbling in basketball—also contain consistent and repetitive movement patterns. Due to the cyclic structure of those tasks, individuals must continuously track the interval since the last sensory and motor events, as well as anticipate the timing of the following [4]. Similarly, in ensemble music performance, musicians must preserve fine temporal regularity while handling intricate visual and auditory input [5,6] and safety-critical work, such as driving or aviation, also involves rhythmic control of motor output under shared attention [7]. Thus, understanding the robustness and vulnerability of sub-second motor timing under cognitive load has both applied and theoretical importance.

Finger tapping, widely used in laboratory settings, acts as a basic experimental model of the described rhythmic activities, primarily to separate and analyze the core timing mechanisms that are the basis for more complex, real-world motor behaviors. Among the numerous variations of that task, probably the most frequently utilized is the synchronization–continuation tapping paradigm, in which participants first synchronize tapping with an external metronome and then continue tapping at the same pace after the metronome stops [5,8]. In human timing research, variability itself is a quantity of interest, as trial-to-trial or interval-to-interval fluctuations in sequential tasks are a key attribute of human timing [9].

Evaluating variability allows us to assess how precisely the brain can represent and reproduce time intervals. Lower variability reflects greater temporal stability, which is why this measure has become central to theoretical accounts of timing. Across the lifespan, timing behavior shows systematic changes that provide context for interpreting variability. As summarized in Bobin-Bègue et al. [10], older adults typically exhibit a slower spontaneous motor tempo (SMT)—interpreted as a deceleration of the internal time base—and tend to produce longer target intervals on time-production tasks. In early development, SMT is faster (IRI ≈ 400–500 ms) at ages 2–7 and gradually lengthens toward ~600 ms by early adulthood, with variability decreasing with age; during synchronization, children show larger IRI variability and often lack the small adult-like negative asynchrony (~10 ms), and they synchronize reliably only near their own SMT, returning to this referent period in continuation [10].

### Automatic vs. Cognitive Timing

It appears that processing intervals below and above one second involve several cognitive and neural mechanisms [11]. Lewis and Miall [12,13] differentiate two distinct timing systems: an *automatic* or implicit timing system that operates over short durations milliseconds to seconds) and a *cognitive* or explicit timing system that operates over longer durations. This distinction is supported by other research as well, including studies by Karmarkar and Buonomano, Rammsayer and Troche, and Herbst et al. [14,15,16]. These systems seem to rely on different neural substrates and cognitive processes. The cerebellum plays a central role in temporal processing, particularly in achieving millisecond precision, potentially through functions of the cerebellar granular layer, which appears to be well-suited for timing operations [17]. On the other hand, cognitive time processing seems to depend on a distributed network involving the frontal cortex, basal ganglia, and cerebellum, with the basal ganglia being implicated in longer interval timing [17,18].

Numerous studies have indicated that, in the suprasecond range, timing is disrupted by concurrent non-temporal tasks [19,20,21], indicating that cognitive timing is more vulnerable to interference. This phenomenon is often studied using dual-task paradigms, which evaluate how performing two tasks simultaneously impacts timing performance [22]. However, it is still uncertain if the same applies to subsecond motor timing, which seems to be more resistant to disruption.

Although specific studies indicate that sub-second timing is mainly *automatic*, several studies suggest cognitive involvement even at these short intervals. For example, Kee et al. [23] discovered that including an anagram-solving task significantly increased variability during a 380 ms finger-tapping task, proposing a reliance on working memory and executive control. In a similar vein, a study by Holm et al. [24] indicated that a significant cognitive demand increased timing variability for both 524- and 733 ms intervals under dual-task conditions. More recently, Mudarris et al. [25] reported that adding a *2-Back working memory task* resulted in increased tapping variability for both auditory cues, more clearly highlighting the role of cognitive resources in fine motor timing.

However, other research opposes this view by demonstrating no reliable interference from a concurrent cognitive task on sub-second motor timing. For instance, Xu et al. [26] found that rhythm-based temporal expectations at a fast tempo (~500 ms) were unaffected by a simultaneous visual working memory task, suggesting that temporal prediction in the sub-second range operates automatically under dual-task conditions. Similarly, Holm et al. [27] reported no significant increase in finger-tapping variability under high executive load when a single hand was used; interference only emerged when motor demands were increased (e.g., bimanual tapping). Other classic studies echo this pattern: Michon [28] and Nagasaki [29] found only marginal dual-task effects on repetitive tapping in the 0.5–2 s range, highlighting the robustness of automatic timing for sub-second intervals. Moreover, Pashler and O’Brien [30] argued that when motor and cognitive tasks can be executed by separate hemispheric systems—such as tapping with one hand and silently rehearsing letters in the other—dual-task interference on response selection is minimized. Finally, Toscano-Zapién et al. [31] examined subtler forms of attentional variation during sub-second tapping. Authors concluded that timing in the subsecond range seems invariant despite the use of different attentional strategies, suggesting a decoupling of automatic timing from cognitive load.

Therefore, although some studies indicate significant dual-task interference, others suggest that sub-second timing remains mostly automatic and unimpacted. To resolve this inconsistency, the present study plans to explore how a simultaneous disrupting task affects the consistency of time intervals during continuation tapping in the sub-second range. Specifically, we will investigate whether the increased cognitive demands placed by a working memory task, known to employ cognitive resources [32,33], will result in quantifiable alterations in the variability of inter-tap intervals, especially the standard deviation of asynchronies, within a 500 ms target interval. This will enable a more precise understanding of the interaction between cognitive load and the automaticity of sub-second timing, possibly uncovering the conditions under which interference effects manifest or are alleviated. The research also makes distinctions between mean asynchrony and the standard deviation of asynchrony as separate measurements of timing performance. This distinction is critical because mean asynchrony shows the average deviation from the target interval. In contrast, the standard deviation of asynchronies quantifies the precision or variability of timing, offering a more nuanced understanding of temporal control.

## 2. Materials and Methods

### 2.1. Participants

The sample was recruited from two local colleges, and the data were collected from 105 students aged 19–25 years (M = 21.8, SD = 1.32). There were 43 males and 62 females. With the sample size, stable estimation in linear mixed-effects models with repeated measures is supported. A post hoc sensitivity analysis using G*Power 3.1 indicated that, given the sample size, medium effect sizes (f = 0.25) could be detected with >0.90 power at α = 0.05 for within-subject contrasts.

Participants were informed about the study procedures and goals and provided written informed consent; they received course credit for participation. During screening, we confirmed that all were right-handed and reported no formal musical training; restricting the sample to non-musicians was intended to reduce variability associated with musical expertise. Other exclusion criteria included a history of neurological or psychiatric conditions and impaired vision or hearing, none of which were reported. The study was approved by the Ethics Committee of the School of Medicine, University of Zagreb.

### 2.2. Tasks and the Procedure

The experiment was performed in a quiet room, and each participant was tested individually after receiving oral instructions. Participants were seated comfortably in an armchair, with their right arm relaxed and resting on a foam arm support that was leveled with a tapping device pad. The hand was positioned palm down at the edge of the arm support so that the index finger, when extended, was above the device pad. The audio stimuli (1000 Hz sinusoidal) were presented via headphones (60 dB SPL) connected to the computer. The FTAP software 2.1.07b [34] was used to run the experiment. As a response input device, an electronic drum pad device (*MIDI Pad Controller Akai Professional MPD218;* stated latency 1–2 ms) was used, as in prior timing studies [9,35].

In both tasks, subjects performed synchronization–continuation tapping with an index finger in inter-stimulus intervals of 500 ms (2 Hz). Participants synchronize with the metronome and perform paced tapping for 20 s. After the metronome signal stops, subjects continue to tap at the given frequency for an additional 32 s. The first four responses were deleted due to adaptation [36], leaving 5–60 taps for analysis.

In the dual-task condition, participants performed the tapping task while simultaneously engaging in a letter-span task designed to impose cognitive load. During the continuation phase of tapping, sequences of eight randomized letters were presented three times at regular intervals, each displayed on the screen for four seconds before disappearing. Participants were instructed to remember the four central letters in each sequence (positions 3–6) and, at the end of the trial, to input the remembered sequences of letters in the presented order using a keyboard. This procedure ensured active engagement with the secondary task. As the study aimed to examine the effect of distraction on timing rather than memory performance, recall accuracy was recorded only as a manipulation check and was not included in the main statistical models. Recall accuracy in the letter-span task confirmed engagement with the secondary task (M = 42.32, SD = 8.34, possible range = 0–60).

We used a letter-span (verbal working-memory) task to impose central attentional load while minimizing sensory/motor overlap with auditory-paced tapping. Auditory pacing offers superior subsecond synchronization and interval discrimination compared to visual/tactile cues [37], consistent with tighter auditory–motor coupling; a visual WM load could introduce sensory-modality competition with the pacing signal rather than isolating mnemonic resources [5,38,39,40].

### 2.3. Measures

As an outcome variable, the duration of the inter-response intervals (IRI) for the continuation phase was recorded by the software. Based on this, the means and standard deviations (SD) of the IRI were calculated, along with the index of accuracy (AI) as a measure of accuracy [41]. The accuracy index is a ratio score that allows one to examine whether individuals tend to under-reproduce or over-reproduce the standards. The nearer the ratio is to one, the more accurate the reproduction. Values greater than 1 indicate reproductions that are longer than the standard, and values less than 1 indicate reproductions that are shorter [41]. The accuracy index was calculated as follows: AI = (Rd − Td)/Td, where Rd and Td represent the participants’ response and target duration, respectively [11,42,43].

### 2.4. Statistical Analysis

We used linear mixed-effects models (LMMs) for three outcomes: inter-response interval (IRI, ms), the standard deviation of IRI (SD), and the accuracy index (AI). Fixed effects were *Condition* (Single vs. Dual task), *Time* (Trials 1–5), and their interaction. Models were fit in SPSS v25 [44,45]. Random effects allowed for subject-specific intercepts and, where justified by fit, subject-specific slopes for *Condition*, to capture individual differences in baseline performance and dual-task susceptibility [46,47,48,49].

LMMs were fit by REML; convergence was verified (alternative optimizer re-fits yielded indistinguishable fixed effects), and retained models were non-singular. Random-slope terms were included only when they improved AIC/BIC and avoided singular fits.

To test whether interference changed over the course of a trial, we analyzed taps 5–60. For each subject and trial, we (a) regressed IRI (ms) on the tap index, with the slope (ms/tap) indexing systematic speeding/slowing, and (b) computed early–late variability as ΔSD = SD_last−SD_first using the first and last quartiles within the 5–60 segment (≥14 taps per window). For each subject and condition, we took the median slope and ΔSD across trials and compared conditions with paired Wilcoxon signed-rank tests.

## 3. Results

### 3.1. Descriptives

Two participants (females) were excluded from the initial sample size, according to standard procedures when utilizing synchronization–continuation timing studies, due to their average interval values being more than 50% above or below the target interval [36,50,51]. Thus, the final sample consisted of 103 subjects.

**Figure 1:**
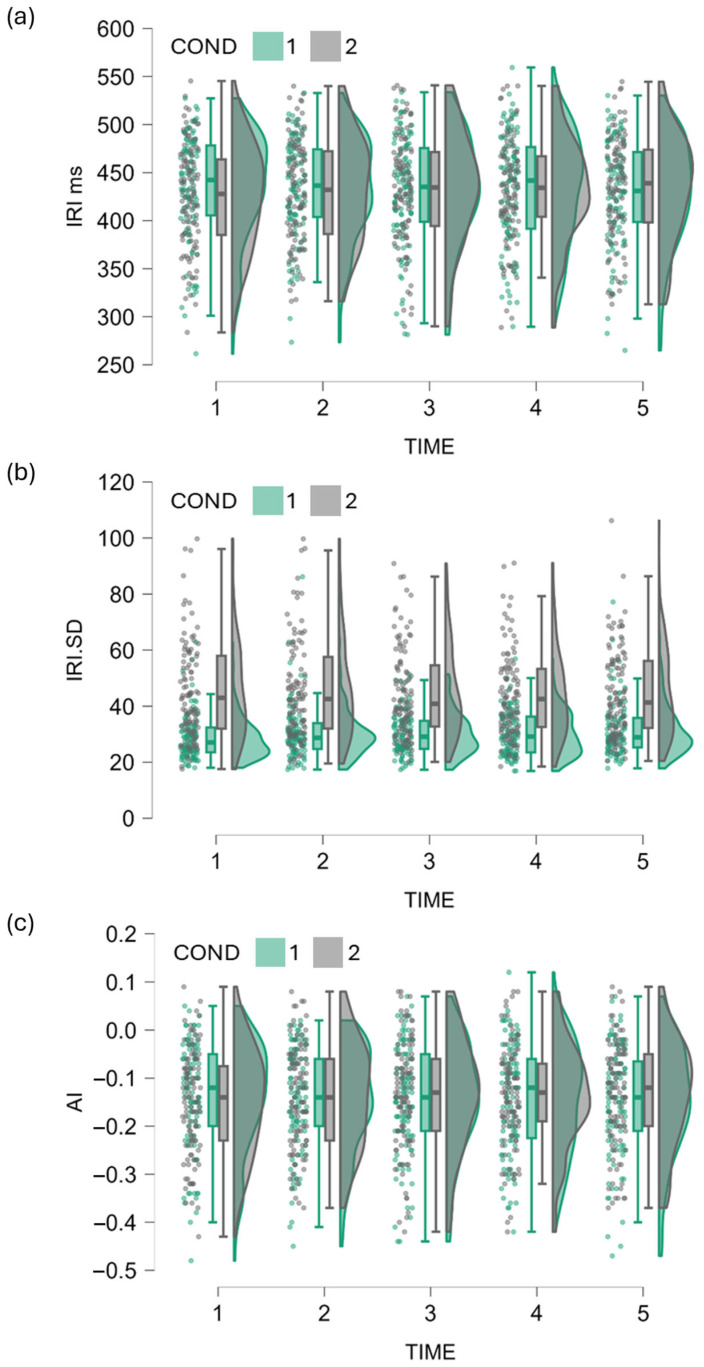
Raincloud plots showing tapping performance across trials (1–5) in single-task (green, COND 1) and dual-task (gray, COND 2) conditions. Each panel displays half-violin densities (kernel estimates) with overlaid boxplots (median, interquartile range, whiskers = 1.5 × IQR) and jittered participant means. (**a**) Inter-response interval duration (IRI, ms) values were shorter than the 500 ms target across both conditions, with only modest trial × condition differences. (**b**) Within-trial variability (standard deviation of IRI, ms) was consistently greater under dual-task conditions, reflecting reduced temporal consistency under increased attentional load. (**c**) Accuracy index (AI) values indicated anticipatory tapping relative to the pacing stimulus, but mean synchronization accuracy was comparable across conditions, with no systematic trial effects.

Descriptive parameters for tapping variables in single- and dual-task conditions are presented in Table 1. Inter-response intervals (IRIs) were consistently shorter than the 500 ms target by approximately 70 ms, suggesting a consistent undervaluation of the target intervals across both conditions. Standard deviations of IRIs demonstrated a significant difference between conditions, with larger variability under dual-task performance. By contrast, accuracy index (AI) values stayed consistent across conditions, consistent with the minimal differences observed in mean IRI duration. Figure 1 provides a complementary visualization of these data, showing distributions, individual values, and condition means, and highlighting both variability and overlap between conditions. Exploratory sex comparisons on aggregated measures conducted separately for single- and dual-task conditions revealed no statistically significant differences (all *p* ≥ 0.18, except for mean IRI in the single-task condition *p* = 0.089).

### 3.2. Linear Mixed-Effects Models

**Figure 2:**
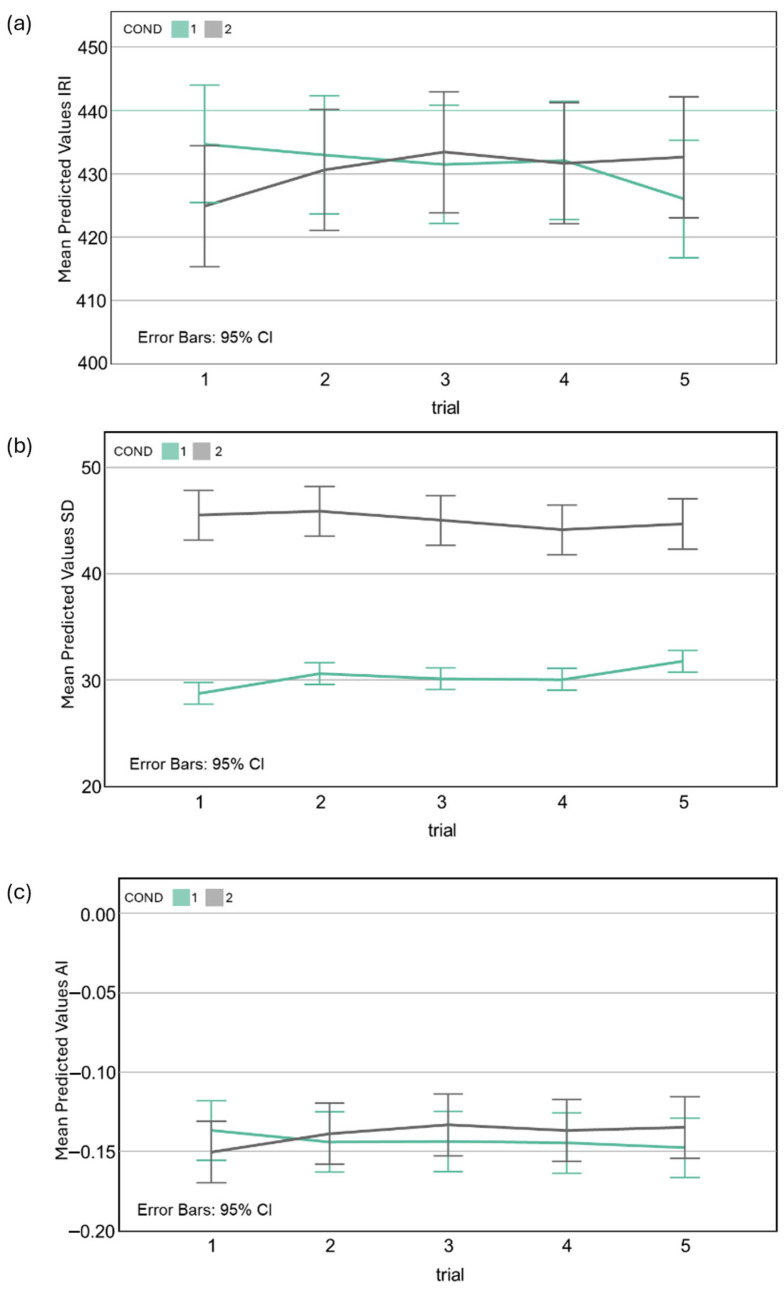
Linear mixed-effects model predictions showing tapping performance across trials (1–5) in single-task (green, COND 1) and dual-task (gray, COND 2) conditions. (**a**) Predicted inter-response intervals (IRI) were consistently shorter than the 500 ms target, reflecting a systematic underestimation of the required timing, but showed only minimal differences between conditions across trials. (**b**) Predicted within-trial variability of IRIs (IRI.SD) was substantially greater under dual-task conditions, indicating reduced temporal stability under increased attentional load. (**c**) Predicted accuracy index values (AI) remained negative, confirming anticipatory tapping relative to the pacing stimulus, but showed no marked differences between conditions or trials. Error bars represent 95% confidence intervals.

To address differences between conditions in the examined variables, linear mixed-effects model analyses were conducted. Model-predicted means with 95% confidence intervals are shown in Figure 2a–c.

#### 3.2.1. Interresponse Interval Duration (IRI)

A linear mixed-effects model (LMM) was adjusted to investigate the effects of *Condition (Single* vs. *Dual task)* and *Time* (*Trials* 1–5) on interresponse interval duration (IRI). Model comparison indicated that the model with random intercepts and random slopes gave a better fit than a random intercept-only model (AIC = 10,060.21 vs. 10,157.68; BIC = 10,074.98 vs. 10,167.52).

The model was estimated using REML and included Bonferroni-adjusted pairwise comparisons. Regarding fixed effects, a significant *Condition × Time* interaction was found (*F* (4, 812) = 2.69, *p* = 0.030). Follow-up tests revealed that the *Single-task* condition had a significantly higher IRI at *Trial 1* compared to the *Dual task* condition (*b* = 16.40, *SE* = 5.21, *t* (812) = 3.15, *p* = 0.002, 95% *CI* [6.18, 26.62]). However, this difference reduced over the subsequent trials (*p* > 0.085). The *Trial 1* difference and the subsequent convergence across trials are visible in Figure 2a. This interaction was driven entirely by *Trial 1*: the dual-task manipulation transiently perturbed tempo on first exposure; from Trials 2–5, mean IRIs did not differ between conditions, and no monotonic *Time* trend was evident within either condition.

Main effects of *Condition* (*F* (1, 101.35) = 0.08, *p* = 0.784) and *Time* (*F* (4, 812) = 0.57, *p* = 0.684) were not statistically significant, suggesting no overall group difference or time trend.

Random effects suggested substantial between-subject variability in baseline IRI scores (intercept variance = 2093.26, *SE* = 329.54), and significant variability in responses to *Condition* (slope variance = 355.32, *SE* = 69.82). The intraclass correlation coefficient (*ICC*) was calculated to be 0.725, indicating that 72.5% of the variance was due to individual differences. Post hoc comparisons confirmed that the IRI was higher under the *Single task* condition at *Trial* 1 (*M* = 434.75, *SE* = 5.54) compared to the *Dual task* condition (*M* = 424.92, *SE* = 5.52). Still, this difference disappeared in later trials (all *p*-values > 0.369).

#### 3.2.2. Temporal Variability (Standard Deviation of IRIs)

To assess temporal variability in motor timing, an LMM was conducted using the standard deviation (*SD*) of IRIs as the outcome. As in the previous analysis, the model included *Condition, Time*, and their interaction as fixed effects, and incorporated random intercepts and slopes for *Condition* at the subject level.

Model fit was improved by the inclusion of random slopes (AIC = 7743 vs. 7967; BIC = 7763 vs. 7981). Variance components indicated meaningful between-subject differences in both baseline variability (intercept variance = 19.12, SE = 11.72) and response to *Condition* (slope variance = 79.37, *SE* = 13.49), with additional residual variance (77.34, *SE* = 3.84). Allowing subject-specific Condition slopes substantially improved fit and converged without singularity, indicating meaningful heterogeneity in dual-task susceptibility.

There was a significant main effect of *Condition*, *F* (1, 99.67) = 117.63, *p* < 0.001: the *Dual task* condition showed higher variability (*M* = 45.06, *SE* = 1.05) than the *Single task* condition (*M* = 30.30, *SE* = 1.06). The elevation in variability under dual-task conditions is evident in Figure 2b. The fixed effect estimate showed that SDs were on average 12.88 ms lower in the single-task condition (*b* = −12.88, *SE* = 1.75, *t* = −7.37, *p* < 0.001).

Neither the main effect of *Time* (*F* (4, 812) = 0.83, *p* = 0.505) nor the interaction (*F* (4, 812) = 1.34, *p* = 0.253) was significant. Bonferroni-adjusted pairwise comparisons also revealed no significant differences across trials.

Slopes of the mean IRI across taps were centered near zero in both conditions (median slope_single = −0.101 ms/tap; median slope_dual = −0.087 ms/tap; Wilcoxon signed-rank W = 2090, *p* = 0.359, N = 99). Early–late variability differences were small in both conditions (median ΔSD_single = +0.74 ms; median ΔSD_dual = +0.58 ms; W = 2335, *p* = 0.652). Thus, the dual-task effect is best characterized as an overall elevation in variability rather than a progressive within-trial drift or buildup.

#### 3.2.3. Accuracy of Motor Timing (AI)

A third LMM was fitted to assess the effects of *Condition* and *Time* on the *Accuracy Index* (AI), a measure of deviation from the target interval. Model comparison showed that the intercept-only model provided a better fit (REML log-likelihood = −1915, AIC = 1911, BIC = 1901) than the model with random slopes (–REML log-likelihood = 2673, AIC = −2667, BIC =−2652). Adding a Condition random slope neither improved AIC/BIC nor avoided near-zero slope variance (singular fits), so a random-intercept-only structure was retained.

The *ICC* derived from variance components was 0.432, indicating moderate individual clustering, with 43.2% of AI variance attributable to between-subject differences.

None of the fixed effects were statistically significant: *Condition* (*F* (1, 918) = 0.78, *p* = 0.378), *Time* (*F* (4, 918) = 0.10, *p* = 0.983), nor the *Condition × Time* interaction (*F* (4, 918) = 0.82, *p* = 0.516). The estimated effect of *Condition* was small and non-significant (*b* = −0.013, *SE* = 0.012, *t* = −1.10, *p* = 0.270), indicating no reliable difference in the accuracy index between the single- and dual-task conditions. The absence of condition and time effects is reflected in the flat trajectories in Figure 2c. Therefore, dual-task performance did not affect AI, and accuracy remained stable across trials and conditions.

## 4. Discussion

The primary aim of this study was to evaluate whether performance indicators in repetitive motor timing are affected by a dual task.

### 4.1. The Negative Mean Asynchrony

The first important observation from the descriptive results is that the accuracy index was negative, indicating that participants, on average, underestimated the target duration by approximately 70 milliseconds in both conditions. However, this discovery is not uncommon and corresponds with earlier research. Aschersleben [52] observed that when individuals are instructed to tap in synchrony with a metronome, they systematically produce their taps just before the beginning of the auditory cue. This systematic tendency to anticipate the beat was named Negative Mean Asynchrony (NMA). According to Aschersleben [52], the taps typically precede the auditory stimulus, on average, for several tens of milliseconds.

NMA is believed to originate from several interrelated cognitive and motor processes, and several theoretical explanations have been proposed for this phenomenon. One key explanation involves perceptual anticipation and time compensation, where the brain predicts the timing of the auditory stimulus, and Aschersleben [52] suggested that this compensation allows the tap to be experienced as synchronized with the tone, although it comes before it. Supporting this view, it was also suggested [5,53] that initial tapping aids in aligning individual experiences with the beat set externally.

An alternative interpretation focuses on predictive timing and internal models. NMA could suggest the function of an internal timing mechanism that predicts when future events are expected to happen, allowing the motor system to plan and carry out the tap before the stimulus arrives. Repp and Su [53] highlight this predictive ability as a central component of sensorimotor synchronization. Moreover, motor control that is specific to the effectors is involved in NMA. Research conducted by Aschersleben and Prinz [54] showed that the degree of negative mean asynchrony can vary depending on the body part employed for tapping—specifically, foot tapping is linked to an increased (more negative) asynchrony compared to finger tapping. This difference can probably be related to differences in peripheral conduction times and the processing of sensory feedback. Likewise, Müller et al. [55] discovered that both finger and toe tapping show significant NMA when subjected to auditory pacing (−40 ms), whereas tapping in response to tactile pacing led to significantly reduced asynchrony (−8 ms). This indicates that the interaction between sensory modality and motor effector additionally affects the degree of asynchrony.

In addition to these factors, the temporal structure of the synchronization task itself influences the magnitude of NMA.

Neuroimaging evidence has also aided in comprehending the neural foundations of sensorimotor synchronization. Using magnetoencephalography, Fukuda et al. [56] examined brain activity during bimanual finger tapping and found differences in sensorimotor cortex activation between alternate and simultaneous tapping modes.

The integration of sensory and motor processes, together with correction mechanisms, is involved in NMA. The brain continuously modifies the timing of actions in reply to sensory input, leading to expected alterations in motor responses. This anticipatory behavior may indicate that a predictive mechanism through which the motor system adjusts for natural neural and motor delays in efferent and afferent pathways, optimizing the temporal alignment between internal motor commands and external events [57]. Consistent with a developmental emergence of anticipatory control, the small adult-like negative asynchrony (~10 ms) is rarely observed in young children [58].

Broadening beyond specific assignments, cognitive–motor synchrony during joint action provides further proof of the resilience of NMA. This occurrence takes place even when people participate in synchronized activities with others, indicating that NMA is based on core principles of sensorimotor coordination. Konvalinka et al. [59] and Miyake et al. [58] demonstrated that, even in complex social and task-driven contexts, synchronization behavior often displays anticipatory tapping features typical of NMA.

Beyond the laboratory, rhythmic synchronization is acknowledged as a critical component of skilled motor performance and is increasingly utilized in rehabilitation [60,61] and sport contexts, i.e., running [62] or soccer [63], where rhythmic synchronization improves both motor precision and adaptability.

### 4.2. Dual-Task Effects on Timing Consistency and Variability

The durations of IRIs demonstrated a short dual-task effect in Trial 1 that vanished through repetition, while the accuracy index remained unchanged. This suggests that dual-task interference mainly affected temporal consistency at the beginning without modifying overall tempo or accuracy.

In comparison, temporal variability (SD) was consistently higher under dual-task conditions, reflecting diminished trial-to-trial precision despite maintained mean timing and AI. This specific influence emphasizes that consistency is more susceptible to cognitive load than global tempo or accuracy [64,65,66]. In other words, dual-task requirements disrupt the predictability of motor timing instead of the ability to sustain a target tempo, a difference that is also highlighted in the research on temporal control [67]. For context, expert musicians typically exhibit lower synchronization variability—and, in some studies, smaller negative mean asynchronies—than non-musicians in standard sensorimotor synchronization tasks; thus, absolute variability levels may differ in expert populations even if the present pattern observed in non-musicians holds [53]. Although our concurrent task was verbal, modality-specific storage is unlikely to be the critical factor: loads that strongly recruit central attention are expected to elevate subsecond tapping variability regardless of code, whereas the auditory advantage and tighter auditory–motor coupling suggest that visual loads may add sensory-competition confounds rather than uniquely verbal effects [5,37,38,39,40,68]. A direct verbal–visuospatial contrast, ideally crossing load modality with pacing modality (auditory vs. visual), would adjudicate modality-specific overlap versus central-load accounts.

Although participants tapped close to their spontaneous motor tempo (SMT), usually about 500–600 ms in young adults [69,70], and SMT is generally regarded as a stable intrinsic rhythm, variability still increased. This indicates that temporal consistency, even at a favorable motor tempo, remains responsive to additional cognitive demands, probably because of disturbances of neural oscillatory processes which underlie predictability [71].

Absolute baselines vary across the lifespan (see Introduction), so the present dual-task increase should be interpreted as an elevation of variability around the young-adult baseline.

This pattern aligns with previous results that cognitive challenges hinder rhythmic consistency [21,24]. For example, Guérin et al. [72] reported that cognitive demand increased finger-tapping variability, especially at fast tempi and with enhanced motor complexity.

In a related study, Wuyts et al. [73] experimentally altered the focus of attention during a bimanual circle-drawing task. They showed that attention influences coordination dynamics directly: patterns needing greater focus became less consistent and more variable, with effects visible at the level of each hand’s timing.

Results obtained in current research support the attentional gate framework [74], stating that when attention is diverted, fewer temporal units are processed, leading to greater variability. The persistence of this effect across trials emphasizes limited capacity for simultaneous timing and executive control [75,76].

Although the observed variability increase (~12–15 ms) is modest, even minor variations can be significant in practice. In ensemble music, timing drifts degrade synchrony and cohesion [6,77]; in sport, they may alter coordination and responsiveness under pressure [3]; and in safety-critical domains, variable rhythmic control can undermine precision and safety [7].

More broadly, the results show that the internal “sense of time” is not fully robust against competing demands. Increased cognitive load makes temporal representations less precise, even when mean accuracy is preserved [78,79]. This supports the view that timing processes, often considered automatic, are tightly coupled with higher cognitive functions [80] and limited by the strategic distribution of scarce attentional resources [81]. Tap-by-tap checks (taps 5–60) showed near-zero slopes and trivial early–late SD changes in both conditions, indicating an overall variability elevation under dual task rather than a within-trial build-up. Taken together, the selective increase in IRI variance (with preserved mean and no within-trial drift) indicates that subsecond timing is efficient but not encapsulated: an automatic generator maintains the mean pace, whereas shared central resources suppress noise via error correction; under concurrent load the noise floor rises, inflating variability without bias.

As an existence proof, in independent single-task data acquired, a Wing–Kristofferson decomposition successfully separated clock and motor variance [82], underscoring that the present paradigm supports formal source partitioning—an important direction for dual-task designs. The mean-tempo effect was confined to Trial 1, consistent with a brief practice/task-set initialization rather than a strategy shift, and aligns with our tap-by-tap checks showing no within-trial drift.

### 4.3. Theoretical Integration and Practical Implications

Together, these results lend support to dual-process accounts of motor timing, which propose somewhat independent neural mechanisms for maintaining temporal precision and average tempo [83,84]. The dual-task expenses were noted in variability, but not in average duration or accuracy, implying that these subcomponents of timing might have varying levels of vulnerability to disruption.

These findings may have real-world relevance for motor performance in domains such as sport, music, or rehabilitation, where individuals often need to maintain precise timing under cognitive load or distraction. In applied contexts, rhythmic cueing can stabilize timing: in a randomized gait-training study, Thaut et al. [61] used rhythmic auditory stimulation (RAS; metronomic pulses embedded in music) and observed considerable improvements in cadence and velocity, along with reduced stride-time variability compared with self-paced or no-training controls. Similarly, in Parkinson’s disease, Hausdorff et al. [85] demonstrated that RAS reduced stride-to-stride variability and promoted more automatic gait, independent of speed changes. It should be noted, however, that our results are based on healthy young adults, and direct generalization to clinical or aging populations should be made with caution.

Therefore, in rehabilitation, improving anticipatory timing is crucial for restoring function [86]. Impaired interval timing, as seen in Parkinson’s disease, contributes to deficits such as bradykinesia and dysmetria [87], highlighting the clinical importance of examining these processes. Moreover, rhythm-based therapies show how the brain’s natural capacity for synchronization can be utilized to enhance coordination and motor control [88].

More broadly, anticipatory capacity in motor timing depends on experience and training [89] and is reinforced by synchronizing with the rhythms of the environment, which refine predictive control and coordination [90]. These anticipatory modifications facilitate everyday tasks, from walking to ensemble music [6,77], and support skilled motor performance in sport [3]. Notably, athletes frequently coordinate rhythmic movements while processing tactical information or responding to unpredictable stimuli—situations similar to dual-tasking. Likewise, in music, keeping a steady tempo during sight reading requires concentrated attention and strong predictive timing [91].

Taken together, these applications illustrate how the dual-task costs observed in the current research may extend to real-world contexts, establishing a basis for both theoretical refinement and applied interventions.

### 4.4. Limitations and Future Directions

Some limitations should be noted. While variability increased robustly, no systematic changes were observed in mean IRI or AI. A further limitation is that the study relied on a single measurement session. Although this is standard in sub-second timing research, it restricts the ability to directly evaluate retest reliability or long-term learning effects. In the present design, learning across five trials within the session could be examined, but future work should incorporate repeated testing across separate sessions to more fully address stability over time. More demanding secondary tasks, or tasks varying in modality, may reveal broader effects. Additionally, although the variability increase was modest, its practical implications should be evaluated in ecologically valid contexts where precision is critical. Finally, individual-difference measures should be incorporated to clarify mechanisms underlying susceptibility to interference [92,93]. Furthermore, the present sample consisted exclusively of healthy young adults with university education. While this is standard in laboratory timing research, it limits direct comparison with clinical or aging populations, such as patients with neurodegenerative diseases, where motor and cognitive loads interact differently. Future work should extend these paradigms to more diverse and clinically relevant cohorts to assess generalizability.

## 5. Conclusions

This study shows that dual-task conditions selectively increase variability in sub-second rhythmic tapping, while mean timing and synchronization accuracy stay consistent. This separation implies that participants depended on compensatory methods to maintain essential timing objectives even as trial-to-trial consistency decreased under cognitive load. Notably, an increase in variability occurred even under favorable tempo conditions, suggesting that temporal consistency remains affected by additional cognitive challenges. These patterns correspond with the view that sub-second timing is tightly coupled with higher cognitive functions but constrained by the strategic allocation of limited attentional resources [80,81]. Findings of inter-individual variability in both intercepts and slopes further highlight the value of modeling individual differences with linear mixed-effects models when analyzing dual-task effects in motor timing [94]. Although the variability increase (~12–15 ms) was modest, even minor reductions can be significant in practical applications where rhythmic precision is critical—music, sport, and safety-critical multitasking [7,95]. *Timing survives distraction, but precision pays the price.*

## Figures and Tables

**Table 1:** Descriptive parameters for outcome variables.

	Condition	N	Mean	SD	SE
IRI	ST	103	432.29	54.90	2.43
	DT	103	430.65	54.17	2.39
IRI.SD	ST	103	30.33	8.70	0.39
	DT	103	45.06	16.53	0.73
AI	ST	103	−0.143	0.111	0.0049
	DT	103	−0.138	0.108	0.0048

IRI-duration of IRIs in ms; IRI.SD-standard deviations of IRIs; AI-accuracy index; ST/DT-single/dual task.

## Data Availability

The raw data supporting the conclusions of this article will be made available by the author on request.

## Abbreviations

The following abbreviations are used in this manuscript:

IRI	inter-response intervals
AI	index of accuracy
LMM	linear mixed-effects model
RAS	rhythmic auditory stimulation
NMA	negative mean asynchrony
SMT	spontaneous motor tempo
